# Rhesus macaque social functioning is paternally, but not maternally, inherited by sons: potential implications for autism

**DOI:** 10.1186/s13229-023-00556-3

**Published:** 2023-07-21

**Authors:** Joseph P. Garner, Catherine F. Talbot, Laura A. Del Rosso, Brenda McCowan, Sreetharan Kanthaswamy, David Haig, John P. Capitanio, Karen J. Parker

**Affiliations:** 1 Department of Comparative Medicine, Stanford University, 300 Pasteur Drive, Edwards R348, Stanford, CA 94305-5342 USA; 2 Department of Psychiatry and Behavioral Sciences, Stanford University, 1201 Welch Road, MSLS, P-104, Stanford, CA 94305-5485 USA; 3 California National Primate Research Center, 1 Shields Ave., Davis, CA 95616 USA; 4 Department of Psychology, Florida Institute of Technology, 150 W. University Blvd., Melbourne, FL 32901 USA; 5 Department of Population Health and Reproduction, School of Veterinary Medicine, University of California, 4205 VM3B, Davis, CA 95616 USA; 6 School of Mathematical and Natural Sciences, Arizona State University West Campus, 4701 W. Thunderbird Rd., Glendale, AZ 85306 USA; 7 Department of Organismic and Evolutionary Biology, Harvard University, 26 Oxford St., Cambridge, MA 02138 USA; 8 Department of Psychology, University of California, 1 Shields Ave., Davis, CA 95616 USA

**Keywords:** Autism spectrum disorder, Autistic traits, Heritability, Parent of origin effect, Primate model, Rhesus macaque, Social functioning, Social Responsiveness Scale

## Abstract

**Background:**

Quantitative autistic traits are common, heritable, and continuously distributed across the general human population. Patterns of autistic traits within families suggest that more complex mechanisms than simple Mendelian inheritance—in particular, parent of origin effects—may be involved. The ideal strategy for ascertaining parent of origin effects is by half-sibling analysis, where half-siblings share one, but not both, parents and each individual belongs to a unique combination of paternal and maternal half-siblings. While this family structure is rare in humans, many of our primate relatives, including rhesus macaques, have promiscuous breeding systems that consistently produce paternal and maternal half-siblings for a given index animal. Rhesus macaques, like humans, also exhibit pronounced variation in social functioning.

**Methods:**

Here we assessed differential paternal versus maternal inheritance of social functioning in male rhesus macaque offspring (N = 407) using ethological observations and ratings on a reverse-translated quantitative autistic trait measurement scale. Restricted Maximum Likelihood mixed models with unbounded variance estimates were used to estimate the variance components needed to calculate the genetic contribution of parents as the proportion of phenotypic variance (σ^2^_P_) between sons that could uniquely be attributed to their shared genetics (σ^2^_g_), expressed as σ^2^_g_/σ^2^_P_ (or the proportion of phenotypic variance attributable to genetic variance), as well as narrow sense heritability (h^2^).

**Results:**

Genetic contributions and heritability estimates were strong and highly significant for sons who shared a father but weak and non-significant for sons who shared a mother. Importantly, these findings were detected using the same scores from the same sons in the same analysis, confirmed when paternal and maternal half-siblings were analyzed separately, and observed with two methodologically distinct behavioral measures. Finally, genetic contributions were similar for full-siblings versus half-siblings that shared only a father, further supporting a selective paternal inheritance effect.

**Limitations:**

These data are correlational by nature. A larger sample that includes female subjects, enables deeper pedigree assessments, and supports molecular genetic analyses is warranted.

**Conclusions:**

Rhesus macaque social functioning may be paternally, but not maternally, inherited by sons. With continued investigation, this approach may yield important insights into sex differences in autism’s genetic liability.

## Introduction

Autism spectrum disorder (ASD) is diagnosed on the basis of two core symptom domains: persistent social interaction and communication difficulties and the presence of restricted, repetitive behaviors [1]. ASD represents the quantitative extreme of autistic traits, which are common and continuously distributed across the general human population [2]. Quantitative autistic traits are highly heritable, both in relatives of autistic probands (i.e., the ‘Broad Autism Phenotype’) [3–6], and in members of the general population [7–9]. This continuum of autistic traits is thus thought to arise from additive genetic susceptibility [10, 11].

Patterns of autistic traits within families suggest that more complex genetic mechanisms than simple Mendelian inheritance—in particular, parent of origin effects—may be involved [12]. However, these complex patterns of inheritance remain poorly understood. This is because discerning parent of origin effects is exceedingly difficult in socially monogamous breeding systems where genetic relatedness and shared (vs unshared) environments are typically confounded (i.e., full-siblings are more likely to share an environment than half-siblings). In theory, some of these confounds could be addressed by using a parent–offspring regression approach to estimate genetic contributions to quantitative autistic traits. However, in practice, this approach is thwarted when mothers carrying ASD susceptibility genes show mild (or no) quantitative autistic traits, but the sons who inherit them do [13, 14].

The ideal strategy for ascertaining parent of origin effects would be to estimate genetic contributions to social functioning in a population of paternal half-siblings and maternal half-siblings, where paternal half-siblings do not share a mother (and vice-versa) [15]. While this experimental design is not feasible in humans, many of our primate relatives have promiscuous breeding systems [16] which consistently produce both paternal half-siblings and maternal half-siblings for a given index animal. Furthermore, individual differences in social functioning (measured by: observation of non-social behavior and social behavior such as grooming and play in the home corral; assessment of face recognition ability and species-typical responses to conspecific social signals in the laboratory), as well as autistic-like trait burden (measured using a reverse-translated rating scale; see below), have been documented and extensively studied in promiscuously breeding rhesus macaques [17–28], making them an ideal study species for such an investigation.

The present study therefore tested for differential paternal versus maternal inheritance of social functioning in a sample of male rhesus monkeys, while avoiding the confounds that have impeded such efforts in humans. Male monkeys were chosen to study in our larger research program because of ASD’s male-biased prevalence [29], and, here, due to availability of their archived behavioral data. To perform these analyses, we exploited methods typically employed in farm animal breeding to identify and quantify: parent of origin effects for phenotypic traits in offspring; parents with particularly strong genetic contributions; and the degree to which parental genetic contributions were evenly distributed across the population versus concentrated in a few individuals. These methods have distinct advantages over methods typically used in human subjects research (including removal of the influence of genetic, environmental, epistatic, or epigenetic confounds), but require more controlled environments and pedigrees [15].

Because each son belonged to a group of paternal half-siblings and to a group of maternal half-siblings, we could estimate the genetic contributions of the father and mother to each son’s phenotype without knowing parental phenotypes. This had two advantages. First, because mothers’ phenotypes were never measured, we circumvented the confound that females (but not their sons) show lower phenotypic expression of genetic susceptibility [13, 14]. Second, these calculations for fathers were controlled for mothers and *vice-versa*, which prevented any shared environmental effects of the father and mother masquerading as genetic effects [30].

We used two statistically correlated [19], but methodologically distinct, social functioning measures in this study: (1) an ethological assessment of the frequency a monkey is observed alone, in non-social behavior [17, 20, 23]; and (2) a rating assessment of a monkey’s quantitative autistic-like trait burden (the macaque Social Responsiveness Scale-Revised [mSRS-R] score) [18, 19, 22]. (The mSRS-R was reverse-translated from a clinically relevant instrument, the Social Responsiveness Scale, which is used in humans to assess autistic traits and to screen for ASD [31, 32]). We then used multigenerational pedigree records to identify in our dataset paternal half-siblings and maternal half-siblings as further detailed below.

## Methods

### Subjects and study site

Subjects were a total of *N* = 407 male rhesus macaques (*Macaca mulatta*) aged 1.17–20.7 years (mean ± SD = 3.30 ± 1.67). Subjects had been born and reared at the California National Primate Research Center (CNPRC) in Davis, CA. Subjects lived outdoors in any one of the 24 half-acre (0.2 ha) field corrals. Each corral measured 30.5 m wide × 61 m deep × 9 m high and contained up to 221 animals of all ages and both sexes. Subjects were tattooed as infants and dye-marked periodically to facilitate easy identification for husbandry- and research-related procedures. Monkeys had ad libitum access to Lixit-dispensed water. Primate laboratory chow was provided twice daily, and fruit and vegetable supplements were provided weekly. Various toys, swinging perches, and other forms of enrichment were provided in each corral, along with outdoor and social housing, thereby providing a stimulating environment.

The present investigation collated behavioral data obtained previously from five study cohorts (referred to below as Cohorts 1, 2, 3, 4, and 5). Subjects in these study cohorts had been selected independent of genetic relatedness, on the basis of the following criteria: male, socially housed in outdoor field corrals, medically healthy, not simultaneously enrolled in another CNPRC project, and previously enrolled in CNPRC’s BioBehavioral Assessment Program [33] as infants.

### Reproductive management and parentage confirmation

The CNPRC houses approximately 4000 rhesus monkeys. A center-wide reproductive management plan has been in place for more than three decades to ensure an outbred colony. The formation of new corrals occurs regularly, and animals from multiple corrals are often combined to further prevent inbreeding. These decisions are guided by a geneticist. It was thus critical to determine the genetic parentage of subjects, which was accomplished using an established panel of microsatellite markers designed to identify maternity and paternity [34, 35].

### Rank ascertainment

An individual’s rank may impact social behavior in nonhuman primates [36]. We therefore included subjects’ rank in the present study, and used the rank information that most closely corresponded to each subject’s behavioral data collection period (see below). CNPRC behavioral management personnel assess monkey ranks in each corral by recording aggressive and submissive interactions following food provisioning. Rank is ascertained on an approximately monthly basis beginning when animals are 2–3 years of age. Because each corral contains a different number of animals, rank is calculated as the proportion of relevant animals in the group that the focal individual outranks, such that the highest-ranked individual has a value of 1 and the lowest-ranked individual has a value of 0 [37]. Rank of course can impact young animals under CNPRC’s age threshold for ascertainment. Rhesus macaques maintain a despotic linear hierarchy [38], and early in life infants assume the rank of their mothers. Thus, for all subjects too young to receive a rank by behavioral management, we assigned the mother’s rank to these subjects. In Cohort 1, all subjects were old enough to have been assigned their own rank in the male hierarchy, whereas in Cohorts 2–5, a subset of subjects were young enough to still retain their mother’s rank. This necessitated that we calculate the proportion of individuals outranked slightly differently between study cohorts to ensure the most accurate ascertainment of a subject’s rank within them. Rank was accordingly Z-scored within each study cohort for use in the present study.

### Behavioral observations and non-social equivalence score calculation

Unobtrusive behavioral observations had been previously conducted on *N* = 376 subjects from four of the five study cohorts (i.e., Cohorts 1, 2, 3, and 4) while they were in their home field corrals as previously described [19, 20, 23]. For Cohorts 1, 3, and 4, observers conducted 10-min focal samples on subjects during two observation periods per day, 4 days per week, for 2 weeks. The behavior of individual monkeys was recorded at 30-s (Cohort 1) or 15-s (Cohorts 3 and 4) intervals using instantaneous sampling. For Cohort 2, we adopted a scan sampling approach, enabling us to score multiple animals in the same group at the same time. Each observer conducted scan samples for a given corral during two observation periods per day. In each observation period, scan sampling was conducted at 20-min intervals, at a rate of 18 scans per day, for a total of five days. During each scan, the subjects in each corral were identified, and observers then recorded the behavior. The same five social behaviors were recorded for all study cohorts (i.e., the ethogram was the same regardless of sampling technique): non-social (subject is not within an arm’s reach of any other animal and is not engaged in play), proximity (subject is within arm’s reach of another animal), contact (subject is touching another animal in a nonaggressive manner), groom (subject is engaged in a dyadic interaction with one animal inspecting the fur of another animal using its hands and/or mouth), and play (subject is involved in chasing, wrestling, slapping, shoving, grabbing, or biting accompanied by a play face [wide eyes and open mouth without bared teeth] and/or a loose, exaggerated posture and gait; the behavior must have been deemed nonaggressive to be scored). Both sampling methods estimate the durations of behavior [39]. After completion of data collection, the total frequency of non-social behavior was then summarized across all of the behavior samples collected for each subject. As three of the study cohorts had been observed using instantaneous sampling methods with varying sampling intervals (i.e., Cohorts 1, 3, and 4), and one study cohort had been observed using scan sampling methods (i.e., Cohort 2), we created a “non-social equivalence score” by Z-scoring non-social behavior frequency within each study cohort to enable comparison of animals across different cohorts following [17].

### mSRS-R ratings

mSRS-R scores had been previously obtained on *N* = 264 subjects from three of the five study cohorts (i.e., Cohorts 3, 4, and 5). Observers rated each subject on a 36-item original mSRS [25], which we had modified from a four-point to a seven-point Likert scale (1 = total absence of the trait, 7 = extreme manifestation of the trait) for each item. Prior to final summary, questions written in the infrequent direction were reverse scored such that higher scores always indicated greater impairment. Since only 17 of the original 36 mSRS items exhibited consistent inter-rater and test–retest reliability, we extracted and tabulated ratings for the 17 reliable items, which form the basis of the mSRS-R [19]. Ratings had been obtained using the same scale across study cohorts, so the mSRS-R ratings did not require normalization.

### Data processing and statistical analyses

We first collated all available data for each subject. As noted above, measures that differed in the observation or calculation method between study cohorts (namely non-social behavior score and rank) were Z-scored within cohort prior to analysis, whereas measures that employed the same ascertainment method across study cohorts (namely mSRS-R score and variables such as age) were not Z-scored. A small number of subjects had been studied twice (i.e., they had been members of two different study cohorts). For these animals, we uniformly discarded their earliest data and retained their most recent data for analysis here.

We then used CNPRC’s multigenerational pedigree records to identify the father and mother for each subject. These data included a small number of full-sibling pairs. For our initial analyses, we uniformly discarded the youngest animal of the full-sibling pair to enable retention of one animal (the eldest) for the half-sibling analyses. Using the same pedigree data, we measured the inbreeding coefficient (F) for 40 random offspring as well as both of their parents. We also estimated the coefficient of relatedness (r) between each of these animals with every other animal included in this subset. While F is defined in terms of the probability of identity in state of different pairs of alleles, r measures the probability that alleles drawn at random from the same locus in each of two subjects will be identical by descent [40, 41]. Differences between sires and dams in these measures were tested by Mann–Whitney.

For the two behavior measures (i.e., the non-social equivalence score and mSRS-R score) we then generated two new datasets, and calculated the number of paternal half-siblings and the number of maternal half-siblings for each subject independently for each measure. Subjects with no paternal and no maternal half-siblings were then removed in each dataset. Thus, in each dataset each subject had at least one maternal or paternal half-sibling, and no full-siblings [42, 43].

Restricted Maximum Likelihood (REML) mixed models with unbounded variance estimates were used to estimate the variance components needed to calculate the genetic contribution of parents as the proportion of phenotypic variance (σ^2^_P_) between sons that could uniquely be attributed to their shared genetics (σ^2^_g_), expressed as σ^2^_g_/σ^2^_P_ (or the proportion of phenotypic variance attributable to genetic variance), and narrow sense heritability (h^2^) following [15]. The individual genetic contribution of a parent is given as Best Linear Unbiased Predictors (BLUPs), generated in the same mixed models [15].

Different genetic scenarios have different dynamic ranges and expected values for σ^2^_g_/σ^2^_P_ [15]. For instance, σ^2^_g_/σ^2^_P_ in half-siblings given unimprinted autosomal effects has a maximal value of 25%, but imprinted genes (which are selectively silenced when inherited from the mother or father) increase σ^2^_g_/σ^2^_P_ for one parent and decrease them for the other. Furthermore, variance estimates for mothers and fathers can be tested against each other. Thus, σ^2^_g_/σ^2^_P_ captures more information than a traditional estimate of narrow-sense heritability (h^2^), despite the two measures being mathematically related [44, 45]. Absolute values of h^2^ are influenced by a wide range of effects, which combined with its strict definition in terms of purely additive haplotypic gametic contribution, limit interpretation. By focusing on additive genetic variance, and gametic potential, h^2^ essentially assumes that allele–allele interactions (e.g., dominance), gene–gene (e.g., epistatic) interactions, and gene–environment interactions (e.g., phenotypic plasticity) are not contributing to estimates of genetic variance. However, this is rarely the case, and so dominance and epistatic effects tend to inflate h^2^, whereas phenotypic plasticity can inflate or deflate it, depending on study design [42, 43]. Furthermore, h^2^ does not imply genetic determinism [42]: for example, h^2^ is often far higher than concordance rates [43], and often exceeds its theoretical limit of 1. Accordingly, we primarily present the results as σ^2^_g_/σ^2^_P_, which is broader but more meaningful in interpretation, has distinct meanings within its dynamic range, and cannot exceed its theoretical limits.

A critical advantage of this approach is that potential confounding environmental effects can be included in the model, and the variance components representing the half-sibling group (i.e., father or mother), are estimated after these confounds are taken into account (i.e., they are the estimate of the unique variance that cannot be explained by other terms in the model [30]). All mixed models included rank, age, and number of males in the social group to control for these potential influences on social behavior. Father and mother were included as random effects to calculate variance components. Furthermore, because each subject (son) belonged to a unique combination of paternal half-sibling group and maternal-half-sibling group, calculating father and mother variance components in the same model eliminated any possibility that shared environmental effects inflated our variance component estimates. Thus, if the son’s social behavior was driven by a shared environmental effect, mother would have no unique explanatory variance once father was taken into account, and *vice-versa* [46]. The power of this approach is that shared environmental confounds are controlled for universally and agnostically even if we do not know what they are [30, 46]. To test whether the variance components attributed to father and mother differed (and, hence, ultimately the σ^2^_g_/σ^2^_P_ and h^2^ attributed to each), we used an F-test of the variance components with their respective degrees of freedom. The variance of σ^2^_g_/σ^2^_P_ was calculated and used to calculate a Z-score and associated *P* value following [15]. Given the relationship between the σ^2^_g_/σ^2^_P_ and h^2^, h^2^ shares the same Z-score and *P* value.

Best practice in linear (and mixed) model design is to stress-test the models to detect potential confounding effects (i.e., “orthogonality checks” or “sensitivity analysis” [46, 47]). In this case, we wanted to ensure the variance attributed to fathers and mothers in the analysis above held up when paternal and maternal half-sibling groups were analyzed separately. For these secondary analyses, the datasets for each social behavior measure were further subdivided and trimmed into datasets where every subject had at least one paternal half-sibling, or at least one maternal half-sibling, respectively. The same mixed models as described above were used, but now with only father or mother included. Processing of the variance components to σ^2^_g_/σ^2^_P_, h^2^, and their tests was performed as described above.

In the non-social equivalence score and mSRS-R datasets, 11 (4.0%) and 7 (3.6%) of the subjects, respectively, were not raised by their birth mother (e.g., because of kidnapping), yielding a total of N = 12 unique animals. To ensure that these subjects were not introducing an artefact, we repeated the analyses above excluding these individuals. Doing so did not change the results. We therefore present the analyses from the full data sets, especially as doing so is the conservative biological and statistical approach (i.e., retaining these subjects should, if anything, reduce the impact of environmental confounds on the final results).

Finally, given the apparent selective genetic contribution from fathers but not mothers, we generated a trimmed dataset for full-siblings. Full-siblings are rare given the rhesus monkey’s promiscuous breeding system. Nevertheless, we were able to analyze full-sibling data for one of our measures: the non-social equivalence score (*N* = 24). The same mixed models were used to generate variance components, σ^2^_g_/σ^2^_P_, and h^2^. We did not have enough full-sibling pairs to calculate meaningful tests for the σ^2^_g_/σ^2^_P_, but we could test the variance component estimates against those from the half-sibling data. These F-tests enabled us to test whether full-siblings differed in the magnitude of their variance components (and hence σ^2^_g_/σ^2^_P_) from paternal and maternal half-siblings.

σ^2^_g_/σ^2^_P_, h^2^, and variance components are population-level summaries, and are not readily visualized. Therefore, for visualization purposes we calculated the predicted genetic contribution of each father and mother (also referred to as the “breeding value”) as the BLUP, which is given as the deviation from the population average.

Mixed models were performed in JMP 15 Pro for Windows. Further calculations of σ^2^_g_/σ^2^_P_, significance tests of σ^2^_g_/σ^2^_P_, and calculation of h^2^, involved several further steps [15] and were performed in Excel.

Note that this statistical approach is essentially equivalent to an “Animal Model” [30] with a pedigree cut at the parental level. The main advantage of the “Animal Model” is its ability to extract additional information from complex pedigrees by comparing individuals with different relatedness in multiple layers of the pedigree. This advantage, however, is minimal here, as we are only comparing siblings, and almost all are half-siblings. Moreover, the cost of implementing the “Animal Model” here is substantial, as it necessitates a range of requirements and assumptions (e.g., a balanced mixture of half- and full-siblings) that cannot be met by this data set, and carries a risk of false negatives due to confounding variables [30]. We therefore chose to adopt the well-established and simpler statistical approach described above.

## Results

Differences between the F values among the dams and among the sires were not statistically significant (Mann–Whitney test, U = 552, Z = 0.88053, *P* > 0.05). Average pairwise relatedness (r) among dams and among sires were also not significantly different (Mann–Whitney test, U = 575.5, Z = − 0.01841, *P* > 0.05). The average pairwise relatedness among all parents was low, 0.016, consistent with values expected for sixth-degree relatives. Average relatedness estimates of dam-offspring and sire-offspring pairs also did not significantly differ (Mann–Whitney test, U = 2901, Z = 0.19901, *P* > 0.05), indicating that heritability estimates are unlikely to be influenced by differences in inbreeding and relatedness levels from the parents and/or unequal levels of relatedness between the offspring and their dams and sires.

**Fig. 1:**
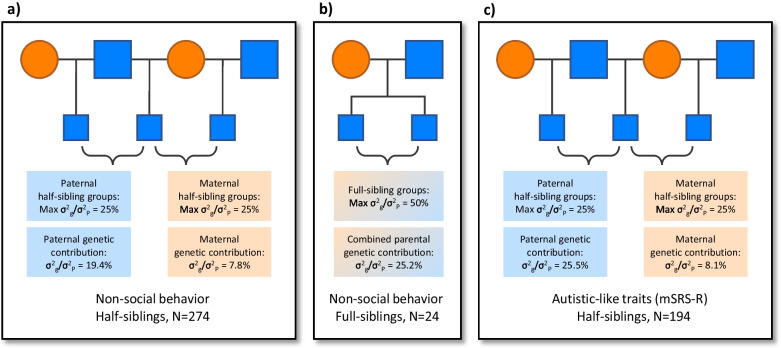
Pedigrees depict the three types of sibling groups and parental genetic contributions to sons’ social functioning. Paternal and maternal genetic contribution was estimated as σ^2^_g_/σ^2^_P_, or the proportion of phenotypic variance attributable to genetic variance. Sons are depicted by small blue squares, fathers by large blue squares, and mothers by large orange circles. **a** Non-social behavior for paternal and maternal half-siblings. **b** Non-social behavior for full-siblings. **c** Quantitative autistic-like traits for paternal and maternal half-siblings. Of the N = 24 full-siblings in panel **b**, the eldest in each pair is included in the half-sibling analysis in panel **a**. Of the N = 274 half-siblings in panel **a**, N = 175 are included in the analysis in panel **c**. Similarly, of the N = 194 half-siblings in panel **c**, N = 175 are included in the analysis in panel **a**. A selective paternal transmission effect was found for both social functioning measures

**Fig. 2:**
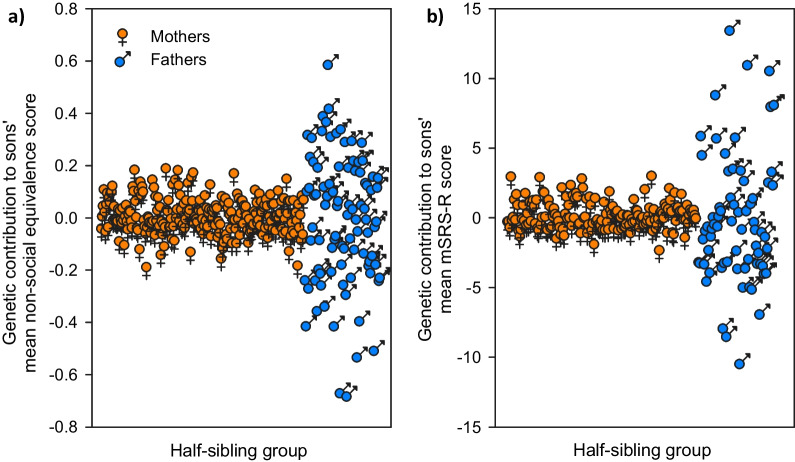
Parental genetic contributions to sons’ non-social behavior and quantitative autistic-like trait variation. The genetic contribution to sons’ **a** non-social scores and **b** mSRS-R scores is estimated as the BLUP for each parent. Each symbol represents the mean deviation of the sons within a half-sibling group descended from an individual parent, plotted on the x-axis in an arbitrary order. The wider range of these values in fathers (blue) versus mothers (orange) is highly significant for each behavior measure and leads to the greater σ^2^_g_/σ^2^_P_ and h^2^ estimates reported in the text. Sample sizes for **a** were N = 89 fathers and N = 237 mothers and for **b** were N = 69 fathers and N = 171 mothers. Non-social behavior was measured using slightly different sampling methods between cohorts and was accordingly Z-scored within each cohort to produce the “non-social equivalence score” shown here, whereas mSRS-R scores were measured with the same scale across cohorts and thus did not require transformation

The main analysis included paternal and maternal half-siblings of each son so that variance components for fathers were controlled for shared environmental effects with mothers and *vice-versa*. For non-social equivalence score, when fathers and mothers were analyzed together (N = 274 half-siblings), paternal genetic contribution was strong (σ^2^_g_/σ^2^_P_ = 19.4%; Z = 2.88; *P* = 0.0040) and highly heritable (h^2^ ± SD = 0.777 ± 0.270); whereas maternal genetic contribution was weak and not significantly heritable (σ^2^_g_/σ^2^_P_ = 7.8%; Z = 0.386; *P* = 0.6997; h^2^ = 0.312 ± 0.808) (Fig. 1a). This difference in genetic contribution between fathers and mothers was highly significant (F_236,88_ = 2.854; *P* < 0.0001), and is evidenced by the BLUPs for fathers versus mothers (Fig. 2a).

We then used “sensitivity analysis”, which is a formal process to rule out false discovery due to subject selection or model design in human medical research [47], thereby ‘stress-testing’ this result. Thus, we calculated variance components treating the subsets of paternal and maternal half-siblings separately. For non-social equivalence score, when paternal half-siblings were considered separately (N = 258), paternal genetic contribution remained significant and highly heritable (σ^2^_g_/σ^2^_P_ = 19.9%; Z = 2.96; *P* = 0.0031; h^2^ ± SD = 0.795 ± 0.269); whereas when maternal half-siblings were considered separately (N = 73), maternal genetic contribution remained non-significant (σ^2^_g_/σ^2^_P_ = 17.3%; Z = 1.08; *P* = 0.2788; h^2^ = 0.694 ± 0.641), confirming a selective paternal effect. Furthermore, for non-social equivalence score, we assessed a small set of full-siblings (N = 24). The maximum expected σ^2^_g_/σ^2^_P_ for full-siblings is 50%. This combined parental genetic contribution was σ^2^_g_/σ^2^_P_ = 25.2% (h^2^ = 0.503 ± 0.553) (Fig. 1b), which was significantly different from the estimates for mothers (F_11,236_ = 4.145; *P* < 0.0001), but not significantly different from those for fathers (F_11,88_ = 1.452; *P* = 0.1644), consistent with a lack of maternal effect and a selective paternal effect.

Finally, we performed the same main and secondary analyses for the mSRS-R score, which showed the same pattern: When fathers and mothers were analyzed together (N = 194 half-siblings), paternal genetic contribution was strong (σ^2^_g_/σ^2^_P_ = 25.5%; Z = 3.08; *P* = 0.0021) and highly heritable (h^2^ ± SD = 1.020 ± 0.331); whereas maternal genetic contribution was weak and not significantly heritable (σ^2^_g_/σ^2^_P_ = 8.1%; Z = 0.314; *P* = 0.7583; h^2^ = 0.323 ± 1.030) (Fig. 1c). Again, this difference in genetic contribution between fathers and mothers was highly significant (F_170,68_ = 3.898; *P* < 0.0001), and is evidenced by the BLUPs (Fig. 2b). Similarly, when paternal half-siblings were considered separately (N = 180), paternal genetic contribution remained significant (σ^2^_g_/σ^2^_P_ = 23.5%; Z = 2.84; *P* = 0.0045; h^2^ = 0.940 ± 0.331); whereas when maternal half-siblings were considered separately (N = 45), maternal genetic contribution remained non-significant (σ^2^_g_/σ^2^_P_ = 0.6%; Z = 0.027; *P* = 0.9782; h^2^ = 0.023 ± 0.835).

## Discussion

Using two methodologically distinct measures of social functioning (i.e., ethological social behavior observations; ratings on a reverse-translated quantitative autistic trait measurement scale), we observed the same consistent and highly significant inheritance pattern in male rhesus monkeys. Specifically, paternal genetic contributions were strong for sons who shared a father, whereas maternal genetic contributions were weak and nonsignificant for sons who shared a mother. This difference between paternal and maternal genetic contributions was highly significant. Corresponding paternal and maternal heritability estimates followed the same pattern. Importantly, these findings were detected using the same scores from the same sons in the same analysis; confirmed when paternal and maternal half-siblings were analyzed separately; and observed in both behavioral measures. Finally, full-siblings showed significantly higher σ^2^_g_/σ^2^_P_ and heritability than maternal half-siblings, but full-siblings did not differ from paternal half-siblings, further suggesting a paternal effect.

These population-level interpretations are supported by the BLUPs for mothers versus fathers. Fathers show a much greater range of BLUPs than mothers for the same sons, which directly leads to differences in σ^2^_g_/σ^2^_P_. Thus, because the same sons are considered, the phenotypic variance to be explained (i.e., σ^2^_P_) is the same for mothers and fathers, but the greater spread in BLUPs for fathers leads to a greater genetic variance (i.e., σ^2^_g_). Furthermore, both fathers and mothers show a unimodal spread in BLUPs, consistent with a polygenic mechanism. If only one or two genes were contributing to this variability, then we would expect to see a clumped distribution of BLUPs. A further important consequence of these findings is that if the lower σ^2^_g_ observed in mothers was simply due to there being more mothers than fathers in the analysis, then the range of BLUPs would have to be equal to or greater than that for fathers. However, the narrower range of BLUPs found in mothers rules out this possibility.

In humans, there is evidence that quantitative autistic traits might be selectively inherited from the father [12], but this cannot be disentangled from the possibility that mothers “mask” their own transmission of autistic traits to sons because of lower phenotypic expression of genetic susceptibility [13, 14]. Because maternal phenotypes were not measured directly here, our findings cannot be due to this phenomenon. Indeed, our analytic strategy was explicitly designed to circumvent this confound in a manner uniquely possible in macaque, and not in human, populations. Thus, these collective findings suggest that social functioning is paternally but not maternally inherited by sons.

Gene-by-environment interactions and incomplete penetrance lower σ^2^_g_/σ^2^_P_ from the theoretical maximum, which makes inferring the mode of genetic transmission here difficult. Nevertheless, the observation that maternal genetic contribution is negligible and significantly lower than paternal genetic contribution (at least for sons), cannot be explained unless Y-linked genes or maternally-silenced autosomal genes are involved. However, the fact that the paternal genetic contribution is less than the maximal theoretical value than would be predicted by Y-linked (100%), or a paternally-inherited imprinted (50%), effects, argues for a complex blend of causal factors, which remain to be identified.

### Limitations

This study had limitations that warrant comment. First, it is important to acknowledge that animal models are approximations that enable study of human phenomena; spending more time alone and having a higher autistic-like trait burden is not the same as having ASD. Thus, how well these monkey findings ultimately generalize to humans, particularly in light of species differences in life history strategies, remains to be determined. Second, paternal half-siblings can be born in the same year, whereas maternal half-siblings are almost always born in different years. There thus may be systematic differences in the temporal structure of paternal versus maternal half-sibling sets which might affect heritability estimates. Third, behavioral measures were not available from parents to further validate these findings. However, if postnatal parent–offspring social interactions were responsible for phenotypic similarities between half-siblings, then we would observe the exact opposite pattern of results, because rhesus monkey fathers engage in little if any parental care [48], whereas rhesus monkey mothers interact closely with their offspring and have a direct impact on their social status [38]. Fourth, these data are correlational by nature, and a larger sample that enables deeper, multigenerational pedigree assessments combined with genetic and epigenetic analyses is now needed. Indeed, this is likely to be a promising avenue for future research, as a recent study using exome sequencing in rhesus monkeys reported preliminary evidence of an association between social phenotypes and specific DNA sequence variants implicated in ASD [28]. Fifth, our full-sibling dataset was small, in keeping with the promiscuous nature of this species, and did not accommodate mSRS-R score assessment. Sixth, our study did not include daughters. Indeed, our research to date has focused on male monkeys due to ASD’s male-biased prevalence [29], the fact that significantly more scientific information was available from male ASD research participants for modeling in monkeys patients’ behavioral symptoms [49], and given females show lower phenotypic expression of genetic susceptibility in the general population [7].

## Conclusions

This study reports that social functioning is paternally but not maternally inherited by sons in a primate species closely related to humans. σ^2^_g_/σ^2^_P_ were roughly similar between full-siblings and half-siblings that only shared a father, providing intriguing support for a paternal origin effect, consistent with [50]. The unimodal distribution of BLUPs suggests that these effects are polygenic and/or epigenetic. Further research is now required to better understand this phenomenon, and with inclusion of daughters, this approach may allow us to investigate in a monkey model causal factors that contribute to sex differences in autism’s genetic liability.

## Data Availability

Data from this study are available upon reasonable request from the corresponding authors (JPG, KJP).

## Abbreviations

σ^2^_g_/σ^2^_P_: The proportion of phenotypic variance attributable to genetic variance
ASD: Autism spectrum disorder
BLUPs: Best linear unbiased predictors
CNPRC: California National Primate Research Center
mSRS-R: Macaque Social Responsiveness Scale-Revised
REML: Restricted Maximum Likelihood
